# Shifting from an individual to an organizational perspective in work environment management – a process evaluation of a six-year intervention program within the Swedish public sector

**DOI:** 10.1186/s12889-023-16059-y

**Published:** 2023-06-08

**Authors:** I Dahlqvist, C Ståhl, J Severin, Magnus Akerstrom

**Affiliations:** 1 Region Västra Götaland, Institute of Stress Medicine, Gothenburg, Sweden; 2 Department of Behavioural Sciences and Learning, Division of Education and Sociology, Linköping University, Linköping, Sweden; 3 School of Public Health and Community Medicine, Institute of Medicine, the Sahlgrenska Academy at University of Gothenburg, Gothenburg, Sweden; 4 Institute of Stress Medicine, Region Västra Götaland, Carl Skottbergs gata 22B, Gothenburg, 413 19 Sweden

**Keywords:** Process evaluation, Workplace interventions, Organization, Work environment, Public sector, Organizational-level intervention

## Abstract

**Background:**

Working systematically with the work environment, particularly the organizational and psychosocial work environment entails several challenges for employers. There is a lack of knowledge on how to best undertake this work. Thus, the aim of this study is to evaluate the process of a six-year organizational-level intervention program where workplaces could apply for additional funds to implement preventive intervention measures, with the intention of improving working conditions and reducing sickness absence within the Swedish public sector.

**Methods:**

The program management process was studied using a mixed-method approach combining qualitative document and content analyses based on process documentation produced between 2017 and 2022 (n = 135), interviews with internal occupational health services professionals in 2021 (n = 9) and quantitative descriptive analyses of submitted applications with decisions from 2017 to 2022 (n = 621).

**Results:**

Qualitative analyses of the process documentation revealed concerns from the project group regarding access to sufficient competence and resources among stakeholders and participating workplaces, and role conflicts and ambiguities between the program and everyday operations. To address these challenges, the application process was developed over time using the knowledge gained from previous years. A change in the mental models in work environment management, from an individual to an organizational perspective, was seen among the project group and the internal occupational health services responsible for implementing most of the granted intervention measures. In addition, the proportion of granted intervention measures on an organizational level increased throughout the years from 39% in 2017 to 89% in 2022. The changes in the application process were believed to be the main contributor to the change among the applying workplaces.

**Conclusions:**

Results indicate that a long-term organizational-level workplace intervention program may be used, by the employer, as a tool for shifting from an individual- to an organizational perspective in the work environment management. However, additional measures on multiple levels need to be implemented to secure a sustainable shift in perspective within the organization.

## Introduction

The organizational and psychosocial work environment plays a vital role in employee health and wellbeing, as well as sickness absence and employee turnover [1–3]. There is also a considerable economic burden for the employers associated with absenteeism (i.e., sickness absence), and employee turnover. However, absenteeism due to health impairments does not just affect the employers’ direct costs. There might also be decreased productivity and performance due to ill health by those employees who remain at work, also known as presenteeism [4]. Over the last decades, there has been a rising challenge with absenteeism and presenteeism among healthcare workers [5–7]. To recruit and retain valuable employees, organizations need to accommodate for the health and wellbeing of their workers. Reducing absenteeism, employee turnover, and presenteeism by improving the working conditions might be a cost-effective way to increase the labor supply of healthcare workers [8, 9]. Working systematically with the work environment, particularly the organizational and psychosocial work environment entails several challenges for employers. There is a lack of knowledge on how to best undertake this work.

To improve working conditions, occupational health interventions on an organizational level have been suggested [10]. These interventions aim to attend to risks associated with physical, psychological, and psychosocial factors through modifying the work environment, where the main focus of these interventions is to change how the work environment and work procedures are organized, designed, and managed [11]. Improving the work environment by targeting the organizational level has further been emphasized by current European legislation [10]. However, although some studies have reported positive effects on employee health using organizational-level interventions [12–14], most studies appear to generate inconsistent and sometimes contradictory results [15–17]. To better understand these inconsistencies, scholars have suggested process evaluations [18, 19]. In addition, combining both qualitative and quantitative approaches to collect data about the process has been suggested [11, 20].

In contrast to evaluations of the intervention outcome, which assess the effectiveness of an intervention through the achievement of goals, process evaluation provides information about the process. Process variables include information about what activities were implemented and under what circumstances, participants’ interaction with the activities and the possible impact of external factors [21]. This leads to further insight into why a complex intervention either was or was not effective. To evaluate this process, the framework of Nielsen and Randall [18] has been suggested. This framework describes process components that may have an influence on the results of organizational-level workplace interventions through three main themes: the context, the intervention design and implementation, and mental models. The intervention design and implementation determines the maximum level of intervention delivery reached, while the intervention context and participants’ mental models can moderate or mediate the link between the intervention exposure and its outcomes. Specifically, how participants understand and practice their formal roles in a given intervention and context is determined by their mental models [18].

Using process evaluations, a wide range of processual factors impacting the effectiveness of organizational-level interventions have been found, including ensuring active engagement and participation among key stakeholders, understanding the situation, ensuring a good fit to the context, aligning the intervention with existing organizational objectives and having a good measure-to-challenge correspondence [18, 22–27]. However, there is still a need for knowledge on how this can be achieved in practice [28].

In 2017, one of Sweden’s administrative regions initiated a large-scale organizational-level intervention program with the intention of improving the work environment and reducing sickness absence among the region’s employees. Within this program, managers, and their Human Resources-support (HR) are able to apply for funds to implement preventive measures on an organizational level. The intervention program has continued over the years with an annual budget of approximately 1.5 million euros allocated by the regional council.

When analyzing the program using data from the first two years of the program, in 2017 and 2018, a discrepancy was found between the challenges which the managers and HR described in the work environment and their suggested measures to resolve them [29]. Consequently, the implemented measures were not able to demonstrate any significant intervention effects on the sickness absence or employee turnover [26]. Since 2019, numerous changes of the intervention program have been made in order to increase the proportion of organizational-level measures and ensure that the proposed measures are aligned with the described challenges. To determine if the changes in the interventional process have contributed to a higher fit to the context, and to identify potential key factors in shifting from an individual to an organizational perspective in the work environment management, there is a need of a long-term process evaluation of the intervention program management process from 2017 to 2022.

This study aims to evaluate the long-term process of a six-year organizational-level intervention program within the Swedish public sector using a three-level framework for evaluating organizational-level interventions, comprising of the intervention contexts, the intervention design and implementation, and participants’ mental models. This will be done by investigating: (1) development of the intervention context from 2017 to 2022, (2) changes to the intervention design and in submitted and granted applications from 2017 to 2022, (3), the influence of the intervention program on mental models among the project group, OH services and workplaces involved in the process.

## Materials and methods

### Intervention design

The intervention program has been thoroughly described before [29], but briefly, managers, together with their HR support were invited to apply for financial resources from the program to implement organizational-level intervention measures, as a complement to the legislated work environment management. These applications were then administered by a specifically created project group consisting of the project owner at the administrative management of the region, representatives from an internal regional research institute with expert knowledge about organizational work environment management, and the internal occupational health services (OH services). Intervention measures from granted applications were implemented by the managers, with or without the support of the internal OH services or external contractors.

### Settings and study population

In Sweden, the healthcare system is managed by three politically elected governing levels: the national level (government), the regional level (21 county councils), and the local level (290 municipalities). The regional level is largely responsible for the funding, planning, and provision of healthcare services [30]. In one of these regions, with approximately 56,000 employees predominately within the healthcare sector (85%), a large-scale intervention program was initiated by the regional concil in 2017. The aim of the intervention program was to reduce the sickness absence among and improve the working conditions for the employees by providing funding for organizational-level intervention measures for individual workplaces within the region. In this region, the individual workplaces were charged on an hourly basis for the assistance from the internal OH services.

During 2017 and 2022, a total of 657 applications were submitted to the intervention program of which 621 were complete (95%) and included in the analyses. The workplaces behind the applications were found in hospital-based healthcare (76%, n = 472), primary care (16%, n = 101), and within maintenance, hospital services or in the culture sector (8%, n = 48). These applications contained in total 746 suggested intervention measures since individual applications may contain more than one measure. Of the suggested intervention measures, 606 (81%) were granted funding, resulting in 504 applications (81% of the complete applications) which were fully or partly granted, Table 1.

**Table 1:** Number of submitted and granted applications and suggested intervention measures, the level of described workplace challenges, and the type of workplace behind the application

	Year						
Descriptive statistics	2017	2018	2019	2020	2021	2022	Total
Submitted applications, n	66	88	71	134	117	145	621
Granted applications, n (%)	53 (80)	58 (66)	62 (87)	107 (80)	100 (85)	124 (86)	504 (81)
Suggested intervention measures, n	93	116	104	167	121	145	746
Granted intervention measures, n (%)	69 (74)	77 (66)	95 (91)	137 (82)	104 (86)	124 (86)	606 (81)
Level of the described workplace challenge							
Organizational, n (%)	90 (97)	113 (97)	103 (99)	163 (98)	114 (94)	144 (99)	727 (97)
Group, n (%)	3 (3)	3 (3)	1 (1)	4 (2)	7 (6)	1 (1)	19 (3)
Individual, n (%)	0 (0)	0 (0)	0 (0)	0 (0)	0 (0)	0 (0)	0 (0)
Type of workplace submitting the application							
Hospital-based healthcare, n (%)	57 (86)	67 (76)	49 (69)	101 (75)	89 (76)	109 (75)	472 (76)
Primary care, n (%)	3 (5)	12 (14)	17 (24)	22 (16)	18 (15)	29 (20)	101 (16)
Maintenance, hospital services or culture sector, n (%)	6 (9)	9 (10)	5 (7)	11 (8)	10 (9)	7 (5)	48 (8)

### Materials and analytical strategy

In the analyses, a mixed method approach was used, combining qualitative document analysis [31], qualitative inductive and deductive content analysis [32], and descriptive quantitative analyses (see below). In addition, multiple data sources were used to gather information on potential changes in the application process and the mental models among those involved in the intervention program. Methodological approaches which use multiple data sources and analytical methods are advantageous because they allow for triangulation, which increases the understanding of the research topic at hand and provides additional insight into the results [33].

The included data sources were (1) generated process documentation from the project group, (2) submitted applications from the workplaces and their corresponding decisions, and (3) interviews with the internal OH services. The model for evaluating organizational-level interventions by Nielsen and Randall [18] was used as a theoretical framework to guide the analysis. This three-level framework specifically for evaluating organizational-level occupational health interventions, comprises of (1) the intervention context, which is divided into the broader organizational and societal (omnibus) context, and the specific (discrete) context in which the intervention is implemented, (2) the intervention design and implementation, and (3) participants’ mental models, i.e., how they conceptualize and understand the issue of interest, and how this understanding influences action or behavioral change.

### Data analysis

#### Changes to the intervention context, design, and mental models within the project group

The intervention process has been documented by the project group, in a total of 250 documents from 2017 to 2022, which were all read through. After exclusion of duplicates and documents which did not contain any relevant or new information, 135 of the documents were included in the analysis. The different documents consisted of meeting notes, evaluations, personal reflections, routines and instructions, PowerPoint presentations and action plans.

To investigate potential changes to the interventional process, i.e. the internal routines for the intervention program, and how they were utilized, from 2017 to 2022, a qualitative document analysis [31] of key concepts regarding the application process and project group was performed where all documents were systematically reviewed by researchers with good knowledge of the organization (ID, MA).

In addition, to identify contextual factors affecting the intervention program, gather information on the intervention design and implementation, and investigate if the intervention program has led to changed mental models among the participants of the project group, a qualitative inductive content analysis [32] was carried out. All documents were read through and coded by two of the authors (ID, MA). The codes were then grouped into categories and potential changes in key concepts within these categories over the years were recorded using the process evaluation model by Nielsen and Randall [18] as a reference point.

#### Analysis of changes in the submitted and granted applications within the intervention program

To investigate whether the proportion of intervention measures at an organizational level and/or the proportion of intervention measures that match the organization’s current challenges had changed during the years, each submitted application between 2017 and 2022 was analyzed using a qualitative deductive content analysis with predetermined categories [32]. The applications were classified according to the level of the challenge described (i.e., individual, group, or organizational) and the corresponding level of the suggested intervention measure [34]. The analysis also included an investigation of the motivations for the suggested measures, including the “measure-to-challenge correspondence” [29]. Thus, the applications were assessed and dichotomized in terms of whether the measures suggested were clearly motivated according to the complexity of the challenges described (yes/no).

The level of challenges described, the suggested intervention measures, and the corresponding measure-to-challenge correspondence were analyzed descriptively for each year together with background factors retrieved from the applications. Potential changes over the years were investigated using Chi-2 tests. Differences between granted and declined intervention measures were tested using Mann–Whitney U tests. Version 25 of IBM SPSS Statistics (IBM, Armonk, New York, NY, USA) was used for all statistical analysis. Statistical significance was set at p < 0.05, and two-sided confidence intervals were used.

#### Analysis of the changes in mental models among the internal OH services

To gain further insights on whether the intervention program has led to changed mental models among those involved in the application and implementation process, nine interviews were conducted in 2021 with representatives from the internal OH services. The interviewees had either participated in the project group, been involved in the implementation of granted intervention measures and/or had worked at the OH services during the intervention program. In the semi-structured interviews, the respondents were asked questions concerning their perception of the region’s preventive work environment management and provision of services within the intervention program and how this work had evolved over time. The interviews comprised of staff with different professions, including occupational health physician (n = 1), occupational health nurse (n = 1), organizational psychologists (n = 2), manager (n = 1), quality developer (n = 1), occupational, health and safety engineer (n = 1) and customer managers (n = 2). The interviews were recoded and transcribed verbatim resulting in about 20 pages per interview (range 14–26 pages). The interviews were analyzed in several steps, by two of the authors (CS, MA) according to principles of qualitative content analysis [35], where a first reading was explorative and served to identify and categorize the experiences from the intervention program and the overall development of a more organization-oriented approach within the OH services. In the second step, the analysis was more directed, using the process evaluation model by Nielsen and Randall [18] as a reference point, where particularly the shift in mental models among the internal OH services staff was described, and how this could be seen in how the staff describe their interactions and work with employers.

## Results

### Development of the intervention context from 2017 to 2022

The qualitative analyses of the project documentation and the interviews with the OH services professionals resulted in a total of six categories which were connected to the intervention context, of which three to the omnibus context (*sufficient knowledge, access to resources*, and *financial conditions*) and three categories to the discrete context (*funds and costs, role and mandates* and *parallel projects*) (Table 2).

**Table 2:** Overview of identified categories, within the intervention context, design and mental models, from the qualitative analyses of process documentation and interviews with occupational health professionals

Themes	Omnibus context	Discrete context	Intervention design	Mental models
**Categories**	Sufficient knowledge	Funds and costs	Application process	Changes in mental models among the project group
	Access to resources	Role and mandates	Project organization	Changes in mental models among OH professionals
	Financial conditions	Parallel projects		Changes in mental models among managers and HR

#### Omnibus context

##### Sufficient knowledge

According to the project group, there has been a general perception of the workplaces not having sufficient knowledge about the organizational and psychosocial work environment over the entire program (2017–2022). Consequently, there were pedagogical challenges and misunderstandings due to the lack of awareness, described as obstacles to the process.

*“[It is] difficult for the workplaces to apply for health promotion on an organizational level when they don’t know what that is - we have to keep an eye out, pick up on it and introduce it” – Project group, 2021.*

Additionally, it was acknowledged that the already strained work environment interfered with what type of intervention measure the workplace wanted and applied for, and that the health promotive perspective was difficult to accomplish due to the larger problems at hand.

A similar lack of knowledge was also described for the internal OH services during the entire program (2017–2022), which required additional support and guidance from the project group.

##### Access to resources

The workplaces have been described by the project group as experiencing a high workload and a lack of resources i.e., time and adequate staffing, since the beginning of the intervention program (2017–2022). As illustrated in the quote below, these pressures sometimes led to workplaces having to postpone or cancel planned interventions, even before the COVID-19 pandemic:

*“Several intervention measures were canceled due to a lack of time (everyday operations got in the way)” – Project group, 2019.*

Both managers and HR have been perceived as busy and difficult to reach by the project group (2017–2022). Consequently, it has been difficult to involve managers in the intervention process.

Based on the meeting notes from the project group, there was a shortage of resources within the internal OH services as well (2018–2022), which sometimes resulted in difficulties delivering the intervention measures in time and the necessity of involving external consultants was discussed. In 2020, there was a change of priorities in both the program and the workplaces due to the COVID-19 pandemic. The project group discussed potential difficulties and insecurities due to these changes:

*“Do the resources at the internal occupational health services constitute an obstacle for continuing to grant and take on more workplaces? There is great uncertainty regarding the demand for their services in the aftermath of COVID-19” – Project group, 2020.*

##### Financial conditions

How services are financed and ordered within the region was identified as an omnibus contextual factor that prevents employers from thinking in terms of organizational-level interventions or requesting such interventions by one of the interviewees within the OH services. Specifically, this related to a system where services are ordered by the hour, disincentivized the ordering of any services not legally required, or which cannot be related to a specific individual case. Thus the studied intervention program was one way of placing focus on organizational issues since it came with external funding for such interventions.

#### Discrete context

##### Funds and costs

For each year, there was a new decision on a budget reinforcement for the intervention program by the regional council. In the first years of the program (2017–2018), the main point of the funding was to reduce sickness absence among employees. However, in the year following there was an increased focus on improving the work environment in discussions within the project group (2019), and in 2020 and onwards this was more explicitly formulated in the assignment description from the regional concil. The workplaces which were granted funding were generally required to carry out the intervention measures during the same year, with some exceptions. For example, some workplaces were able to postpone the implementation of their granted intervention measures due to the COVID-19 pandemic (2020–2021).

At the start (2017–2018), the workplace was responsible for estimating potential costs of the suggested intervention measures in the application, on which the central administration based their decision. However, this was discontinued the following year (2019), and the project group instead submitted a proposal based on the application regarding the allocation of funds to the central administration, who still made the final decision. After approval, the workplace was responsible for carrying out the intervention, and all remaining processes. This was further developed in 2020, where the final decision was taken based on the intervention measure itself, rather than the costs, provided that the interventions were to be implemented by the internal OH services. For external suppliers, the workplace was responsible for estimating the costs and later forwarding the invoice to the project group, if approved.

##### Roles and mandate

The meeting notes from the project group illustrate the challenges of changing roles, poor role definition and unclear mandates over the years (2017–2022). At the start (2017–2018), there were discussions among the project members about the political process and the uncertainty of being able to influence the direction of the intervention program in the region. However, in 2019 it was acknowledged that they had already gained some influence, as described by the project group:

*“What’s happening is that the project group is entering new arenas and having an impact that was not possible before, with the support of the intervention program (more mandate)… We have been given responsibility for distributing the money, therefore we can ask the questions.” – Project group, 2019.*

Nonetheless, the mandate of the intervention program has occasionally been seen as unclear and fluctuating by the project members. Moreover, there have been expressions of role uncertainties and role ambiguity over the years (2017–2022). The project members recognize that it has sometimes been hard for the workplaces to differentiate between their roles in the OH services and the project group. Correspondingly, the workplaces were seen to express frustration about unclear expectations and a lack of information. The project members discussed how this might have influenced the process:

*“We have been too unclear about what we mean and how it is supposed to be done, and it meets a reality where there is often a gap between the workers on the ground, management and HR” – Project group, 2019.*

##### Parallel projects

The project group have been managing different parallel projects within the same political iniative throughout the years (2017–2022). In the beginning (2017–2018), there were two different ways to receive funding. In addition to the application process, the occupational health experts from the internal research institute carried out systematic analyses of all workplaces in the region to identify those with the greatest need for intervention measures. Likewise, there was a similar process added during the COVID-19 pandemic to support organizations most affected by the pandemic (2021–2022).

### Changes to the intervention design and applications from 2017 to 2022

The qualitative analyses of the project documentation and the interviews with the OH services professionals resulted in two categories connected to the intervention design theme (*Application process* and *Project organization*) (Table 2). In addition, quantitative analysis of the submitted applications to the program identified changes in submitted and granted applications to the intervention program throughout the years.

#### Changes in the intervention design

##### Application process

During 2017 and 2018, the workplaces identified needs and designed interventions without support from the project group or the OH services. The managers, with support from their HR staff, then applied for funds to implement the suggested intervention measures using a standardized application form where they were asked to describe the workplace challenges at hand, the suggested intervention measure, and the amount of funding for which they were applying. Based on the application form, the project group either granted or declined funds for the suggested measures. In 2019, a more comprehensive application process was formed, where the managers and their HR submitted a preliminary application and received feedback through a meeting with the OH services to further develop the application and the suggested intervention measures before submission. The feedback meeting was developed further in the years following (2020–2022), and additional knowledge about the workplaces was added to the project group by inclusion of the customer managers from the internal OH services (see below) which enabled the project group to support the workplaces more actively throughout the application process. In 2019 and 2020, the workplaces were able to apply for, and receive, additional support during the process, and develop a strategic group, as well as particpate in educational/training activities. In 2022, the workplaces were no longer able to suggest intervention measures, instead all parts of the design of intervention measures were done in cooperation with the project group and/or the internal OH services.

##### Project organization

The central administration has been both the owner of the intervention program and responsible for making the decisions on the submitted applications during all six years of the program. There has also been an operational group within the project group throughout the years responsible for the practical implementation of the intervention program process. However, the composition and tasks of the project group changed over the years. The first two years (2017–2018) the group was led by occupational health experts from an internal research institute and the internal OH services were described by the project group as a client and resource, to whom knowledge and methods were distributed. From 2019 and onwards, the project group was professionalized with a formal project leader and the main responsibility for the application process was transferred from the internal research institute to the internal OH services. Correspondingly, the role of the occupational health experts from the internal research institute changed, with an increased focus on providing support and guidance to both project members and workplaces (2019–2022). Also starting in 2019, the customer managers of the OH services were themselves included in the process to enable the project group to use the combined information from both the submitted applications and the knowledge of the particular workplace from the customer managers in their decision. In 2019, the project group was also strengthened with a reference group, consisting of representatives from the internal OH services, the research institute, and the joint management board of the research institute and OH services. The reference group was activated during the evaluation phase at the end of each calendar year and during the planning phase for the next year and was responsible for providing the project group with feedback from the operations.

The managers and HR have consistently been acknowledged as responsible for managing the intervention within their workplace (2017–2022). However, their responsibility for independently designing intervention measures has continually decreased each year, as the support from the OH services has simultaneously increased.

#### Changes in submitted and granted applications to the intervention program

Almost all the submitted applications between 2017 and 2022 (mean 97%, range 94–99%) described workplace challenges on an organizational level and the remaining were on a group level, Table 1. The described challenges were highly workplace specific (i.e., contextual) and included, for example, problems with recruiting specialized staff, high sickness absence or staff turnover among employees and managers, increased demands due to reorganizations and/or changes in the need of the workplaces’ clients or patients on an organizational level, and conflicts or destructive cultures on a group level.

**Fig. 1:**
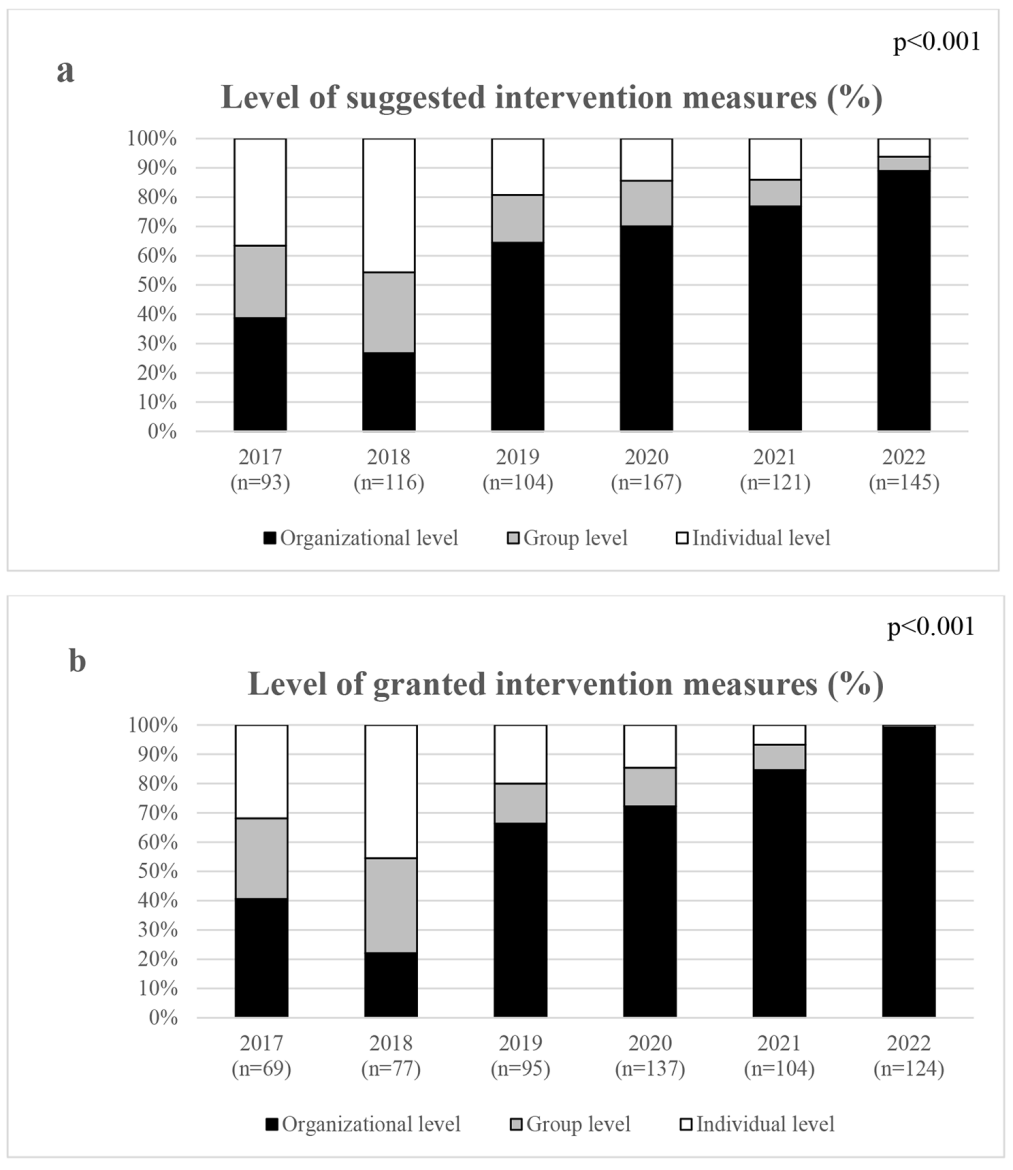
The percentage of suggested (**a**) and granted (**b**) intervention measures on an organizational-, group and individual level, respectively

**Fig. 2:**
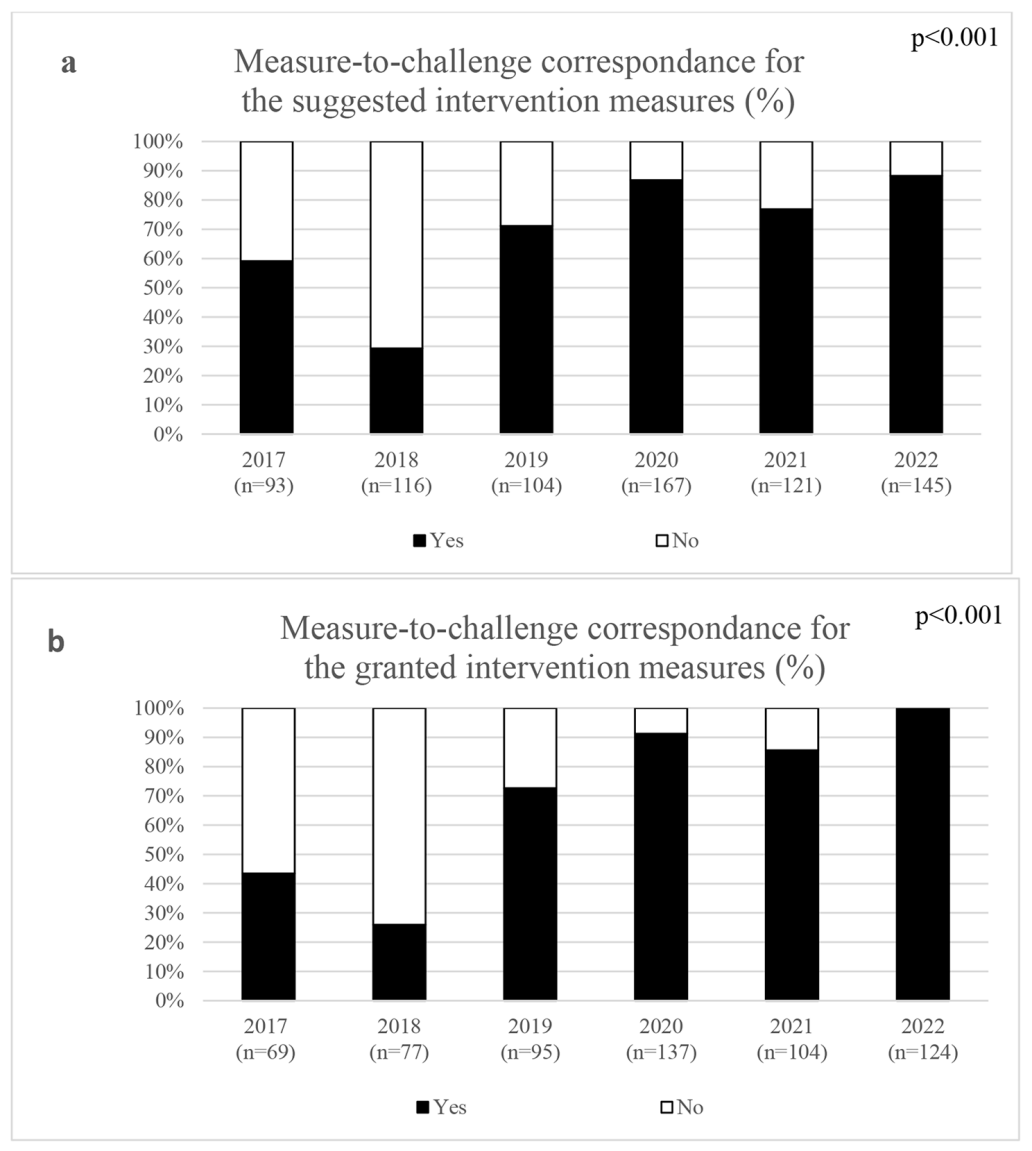
The percentage of suggested (**a**) and granted (**b**) intervention measures with and without a measure-to-challenge correspondence, respectively

When investigating the level of the suggested measures, the percentage of suggested measures on an organizational level had increased from 39% in 2017 to 89% in 2022 while the percentage of suggested measures on a group or individual level had decreased from 25% and 37% in 2017 to 5% and 6% in 2022 (p < 0.001), Fig. 1. Similarly, the percentage of suggested measures with a measure-to-challenge correspondence have increased from 59% in 2017 to 88% in 2022 (p < 0.001), Fig. 2. Even the suggested measures were highly contextual and contained a wide range of different types of measures. In the first years, a relatively high percentage of the managers were applying for funds for motivational speakers, lifestyle interventions, group-based measures aiming to increase the team spirits or improve support within a group of employees, and workshops or staff meetings at external conference centers. The possibility for the latter type of measures was restricted in 2019. However, while these intervention measures were still commonly suggested, a higher percentage of interventional measures aiming to identify root causes of their workplace challenges and/or providing processual support in the change management process could be seen when the managers and their HR received support in the design of interventional measures.

A vast majority of the submitted applications were granted between 2017 and 2022 (mean 81%, range 66–87%), Table 1. Prior to 2020, no difference in the levels of the intervention measures or the measure-to-challenge was seen between the granted and non-granted intervention measures, i.e., the application process did not facilitate that more intervention measures on an organizational level, or measures that were clearly motivated by the workplace challenge were granted. However, from 2020 and onwards, a significantly higher percentage of intervention measures on an organizational level, and a higher percentage with a positive measure-to-challenge correspondence were granted compared to the non-granted intervention measures (p < 0.001, respectively). This increased the percentage of granted measures on an organizational level, and with a measure-to-challenge correspondence to 99 and 100% in 2022, respectively, Figs. 1 and 2.

### The influence of the intervention program on mental models from 2017 to 2022

Using the results from the qualitative analyses of the project documentation and the interviews with the OH services professionals, changes in mental models from 2017 to 2022 were identified, resulting in three categories; *Changes in mental models among; the project group, OH professionals, and managers and HR* (Table 2).

#### Changes in mental models among the project group

Using the project documentation, the development of mental models within the project group were seen both regarding how the intervention measures should be designed and what criteria should be applied. The meeting notes showed that the long-term perspective of intervention measures was somewhat discussed among project members at the start (2017–2018). However, it gained increasingly more attention the years following and resulted in additional requirements in the application process (2019–2022). Furthermore, the importance of a well-founded analysis was first discussed, which then developed into focusing on root causes (2018–2019). Finally in 2020 and onwards, there was an increased focus on the health-promoting perspective and organizational learning, as illustrated in the quote below:

*“The purpose is to get the workplace to think in a different way. To help the workplaces understand what they need…” – Project group, 2020.*

Additionally, the preventive perspective has been a topic of discussion among the project group throughout the years (2017–2022), as well as the shift in perspective from an individual to an organizational level. Similarly, it has been emphasized that the intervention should be seen as a complement to the ordinary operation and not replace any already-planned interventions. Furthermore, at the start of the intervention program (2017–2018), the project members discussed that there should be a fair distribution of the funds, after which a prioritization was discussed to a greater extent, illustrated in this quote:

“*We have a mission from the politicians to improve the work environment, to invest measures and money where it is beneficial. It is not a fairness competition” -Project group, 2019.*

#### Changes in mental models among OH professionals

Development of mental models related to the intervention program could also be seen in the interviews with OH professionals. During the first years there was a strong emphasis on shifting the perspective from the individual to the organizational level when dealing with health issues in workplaces, e.g., through initiatives run from the management and the internal research institute. These included specific educational interventions, not least following a new legal provision from the Swedish Work Environment Authority in 2016 which clarified employers’ responsibilities for the organizational and psychosocial work environment. This provision was recurrently mentioned in the interviews as highly influential, and that it served as a paradigm shift for how to work with health issues; it also legitimized focusing on organizational and preventive approaches. Among the respondents, there was a general sense that the organizational perspective had become well-established among the OH services professionals, and several of the respondents mentioned a shift also in their own thinking, where the individual is now seen as a bearer of organizational symptoms.

*“It’s like a re-programming of the brain” -Occupational health physician, 2021*.

Shifting the perspective lifts the blame and shame from the individual to the organization, and it was argued that this made rehabilitation easier. This shift was generally considered a positive development. Some addressed the risk of a shift of status from those working with individually oriented rehabilitation to those focusing on preventive and organizational interventions (e.g., from doctors to organizational psychologists and other occupations). Overall, however, this was not considered to be a problem by the respondents. While some had experienced this as an issue, it was one that could be managed through dialogue. The OH professionals mentioned the intervention program as being particularly useful in this respect since it involves an extra budget for carrying out interventions in the work environment.

One part of changing the way the OH services work was to introduce more structured systems for how contacts with employers (i.e., their customers) connected to preventive measures should be taken, going through staff with specific customer contact functions. This process also resulted in a more standardized system across the OH units, which some respondents considered to be a negative development, since it reduced flexibility and direct contacts with employers. One of the respondents, a quality developer, considered this more structured process a prerequisite for changing the way the organization works, and therefore also related to the shift toward more of an organization-oriented perspective.

#### Changes in mental models among managers and HR

The shift in mental models that was reported from the project group and the OH services professionals was not necessarily taking place among the managers and HR. These, pointed out by several respondents within the OH services, still wanted and requested individual interventions, which sometimes complicated contacts since.

*“Customers do not get what they think they need” - Organizational psychologist, 2021*

Several respondents discussed how it was a challenge, but a necessity, to be able to talk to employers about what type of support they needed to solve their problems, and that this was now part of their professionalism. Consequently, the OH services professionals could be met with resistance from the workplaces since their suggested solutions were sometimes seen as a criticism of how employers managed their problems.

## Discussion

This aim of this study was to evaluate the process of a large-scale six-year intervention program, where it was shown that directed funds can be a way for employers to increase the number of preventive measures on an organizational level in their systematic work environment management. However, the results suggest that organizational learning is mainly limited to those involved in the implementation of the intervention program and that there are several supporting and hindering factors which affect the implementation and need to be addressed.

In line with previous findings, the analysis of the quantitative data revealed initial challenges with matching intervention measures with described work environment challenges [13, 16, 29]. There was a large discrepancy seen in the earlier years with a poor measure-to-challenge correspondence. However, both the measure-to-challenge correspondence as well as the percentage of measures on an organizational level was significantly increased over time. Several changes in the application process were identified that may have facilitated this change, such as securing sufficient knowledge in the project group, increasing the support to the applying workplaces in identifying intervention measures and stricter criteria in the application process. Previous studies show that support, participation and involvement of managers and employees, functioning communication, as well as education, are important to ensure the development of shared mental models on the current issue and to find appropriate interventions [15, 18, 22, 23, 36]. The involvement and guidance from the project group and OH services with expertise of organizational interventions and the local contexts may have been a success factor in the intervention program. For workplaces which lack the capacity to manage implementation and change, this role is crucial to facilitate interventions with a better measure-to-challenge correspondence [23]. These findings are promising since ensuring a good fit between the intervention measures and the organizational context is crucial to achieve positive and long-lasting intervention effects [18, 22, 24]. Thus, through the multiple changes in the application process, the intervention measures may have been better aligned with existing organizational objectives and structures.

Meanwhile, several obstructing processual factors were found in the intervention context which the project group were not able to address despite the long-term program of six years and the many adjustments in the application process. In addition, the COVID-19 pandemic, during 2020 to 2022, further amplified some of these conditions, although the effects were less than expected. The most salient barriers identified were related to access to sufficient resources and competences among both the workplaces and the OH services and unclear and conflicting roles among the project group and the OH services. Previous studies have acknowledged insufficient resources as a restriction in work interventions [18, 23, 27]. Sufficient time and resources for managers is particularly important since the support and involvement of managers is necessary for a successful intervention implementation [18, 37]. Accordingly, for the managers to be able to engage and work with these processes, active support from top management is essential [15, 37], as well as sufficient strategic resources [23]. Thus, the results emphasize the importance of systematically working with the work environment on all levels as some functioning aspects of the context may be a prerequisite for the process. Moreover, these efforts have been recognized as beneficial to all workplaces regardless of the need for intervention measures [38].

Similarly, a lack of resources was seen within the OH services, which may have further impeded the process. Additionally, how services are ordered and financed was recognized to be an important factor in the preventive work environment management, where a centralization of costs was thought to encourage the use of their services as well as facilitate the cooperation, which is an issue that has been raised in other studies too [39]. In addition, previous studies show that the acceptance of suggested intervention measures depends to a considerable degree on the credibility of those who present it [15]. Mutual trust between OH services and workplaces has previously been acknowledged as key for developing a close cooperation, where close and long-term cooperation serves to build social capital between service providers and customers [39], and may facilitate the readiness to receive support [23].

The findings of this study suggest a change in mental models among both the project group and OH professionals, which points to a positive effect of the intervention program since changes in mental models may result in an increased ability to successfully deal with further challenges independently, i.e., through the development of learning capabilities [22]. Supporting organizations’ capacity to manage change through knowledge building has previously been emphasized, and research shows that workplaces with this capacity achieved larger positive effects of an intervention [23]. These changes were facilitated by a combination of the introduction and development of more structured systems, legal provisions and and experiences gained throughout the intervention program. However, OH professionals did not perceive a similar change of mental models among the workplaces they interacted with, which can be partly explained by the participating workplaces having changed over the years, i.e., most workplaces had a single point of contact with the intervention program and the project group could not follow the development within a workplace over time. In addition, shifting perspective from an individual to an organizational perspective in the systematic work environment management is also a cultural change within an organization and it may take a longer time for potential effects on the mental models to occur. The absence of a change in the stakeholders’ mental models was also found to be an obstructing factor in the process as also seen by others [18, 22, 23]. Thus, additional measures within the entire organization, on multiple levels, need to be implemented in order to not just secure sufficient resources for the targeted intervention program to reduce sickness absence and employee turnover and improve the employees’ working conditions, but also to increase the organization’s capacity to successfully deal with further challenges and enable a shift from an individual to an organizational perspective in the preventive work environment management.

### Methodological considerations

This study contributes with knowledge about process factors affecting the implementation and possibly also the effectiveness of organization-level interventions [15, 18]. Using the theoretical framework by Nielsen and Randall [18] to support this exploration was a strength as it systematically guided the analysis. This study was based on a mixed-method approach and the intervention process was investigated through a combination of quantitative and qualitative data, which offers several advantages [33, 40]. Through data triangulation, it was possible to identify promoting and hindering factors in a complex interplay. Another strength of this study was the access to different perspectives of involved stakeholders by the access to the organization´s own documentation from the project group, submitted applications from the workplaces, and interviews with the OH services. The integration of stakeholder perspectives allows for a richer knowledge of the complex social systems in which these processes take place [40].

However, since the project group’s own documentation was used, rather than structured data collected for the purpose of this intervention program, the amount of documentation and how meetings were documented differed throughout the years, which may be a cause of concern when investigating changes over time. Another limitation of this study was the perspectives of workplaces mainly being limited to second-hand information from other sources, i.e., the process documentation from the project group, submitted applications and interviews with the occupational health professionals rather than interviews with HR and managers. It is also worth mentioning that the workplaces that apply for support experience larger challenges in the work environment, i.e., the barriers described might be present in organizations with large work environment challenges rather than reflecting all workplaces within the organization. Additionally, the interviews with the occupational health professionals were only conducted in 2021 and they were asked to recall changes over time rather than conducting repeated interviews.

## Conclusions

The findings indicate that a long-term organizational workplace intervention program, funded centrally, may be used as a tool for shifting from an individual- to an organizational perspective in the work environment management. The findings of this study suggest a significantly improved measure-to-challenge correspondence over the years as a possible effect of the multiple changes to the application process. Furthermore, the findings imply that the application process was continually developed because of changed mental models among both the project group and occupational health professionals. However, a similar change of mental models was not necessarily seen for the participating workplaces, which could plausibly be due to longer follow-ups being needed to observed these changes. Additionally, there were multiple contextual barriers acknowledged to impede the process, such as limited knowledge about the organizational and psychosocial work environment management within the organization, a lack of resources among managers and the OH services and unclear and conflicting roles within the program that need to be addressed when designing future intervention programs. Thus, the results emphasize the importance of a systematic work environment management which creates sufficient conditions to enable a shift in perspective as well as the need for additional measures on multiple levels to secure a sustainable shift in perspective within the organization.

## Data Availability

The datasets used and/or analyzed during the current study are available from the corresponding author on reasonable request.

## List of Abbreviations

HR: Human Resources
OH Services: Occupational Health Services
